# Network Analysis of Temporomandibular Disorder Pain and Subject‐Based Bruxism in Post‐Traumatic Stress Disorder Patients

**DOI:** 10.1111/joor.14007

**Published:** 2025-05-09

**Authors:** Joey Chung, Wendy Knibbe, Thiprawee Chattrattrai, Ad de Jongh, Frank Lobbezoo

**Affiliations:** ^1^ Department of Orofacial Pain and Dysfunction Amsterdam the Netherlands; ^2^ Department of Masticatory Science Faculty of Dentistry, Mahidol University Bangkok Thailand; ^3^ Research Department PSYTREC Bilthoven the Netherlands; ^4^ Department of Oral Public Health Amsterdam the Netherlands; ^5^ School of Health Sciences, Salford University Manchester UK; ^6^ Institute of Health and Society, University of Worcester Worcester UK; ^7^ School of Psychology, Queen's University Belfast UK; ^8^ Department of Orofacial Pain and Jaw Function Faculty of Odontology, Malmö University Malmö Sweden

**Keywords:** anxiety disorders, bruxism, pain, post‐traumatic stress disorder, self report, temporomandibular joint disorders

## Abstract

**Background:**

Post‐traumatic stress disorder (PTSD) is a psychosocial factor of interest in the multifactorial aetiology of temporomandibular disorder (TMD) pain, awake bruxism (AB) and sleep bruxism (SB).

**Objective:**

To investigate direct and indirect associations between post‐traumatic stress disorder (PTSD), TMD pain, AB, SB and demographic and psychological variables using network analysis.

**Methods:**

The study sample included 597 subjects recruited from a specialised centre for the treatment of PTSD. Network analysis was performed using a Mixed Graphical Model and included variables of self‐reported TMD pain, self‐reported AB, self‐reported SB, age, sex, PTSD symptom severity, mood disorders, anxiety disorders and insomnia severity. These variables were visualised in the network model as nodes connected by edges, representing individual associations.

**Results:**

The network model revealed a triangular positive association between TMD pain, AB and SB. AB also displayed a positive connection with anxiety disorders, while PTSD symptom severity was linked to insomnia, mood disorders and anxiety disorders. Age and sex did not significantly influence the network structure, although a negative association was observed between these variables, indicating younger ages among female subjects.

**Conclusion:**

TMD pain, AB and SB were strongly associated with each other in subjects with PTSD. The presence of anxiety disorders emerged as a bridge factor, connecting the triangular positive association between TMD pain, AB and SB with psychological conditions (PTSD severity, insomnia severity, mood disorders).

## Graphical abstract

This study investigates direct and indirect associations between post‐traumatic stress disorder (PTSD), temporomandibular disorder (TMD) pain, awake bruxism (AB), sleep bruxism (SB) and demographic and psychological variables using a network analysis. The analysis indicates that among subjects with PTSD, TMD pain, AB and SB are strongly associated with each other. Anxiety disorders serve as a bridge factor between AB and PTSD symptom severity in this network.

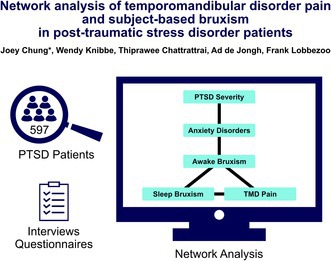

## 1 Introduction

Temporomandibular disorders (TMD) can be described as a group of disorders affecting the masticatory muscles, temporomandibular joints and contiguous structures [1]. TMD can be divided into disorders affecting the temporomandibular joints and disorders affecting the muscles of mastication and may cause pain and disability [2]. TMD pain is considered to be the most prevalent chronic orofacial pain condition [3]. Bruxism is defined as a masticatory muscle activity that is not a movement disorder in otherwise healthy individuals, and it can be divided into two different entities with distinct aetiologies: awake bruxism (AB), characterised by repetitive or sustained tooth contact and/or by bracing or thrusting of the mandible and sleep bruxism (SB), characterised as rhythmic (phasic) or non‐rhythmic (tonic) [4, 5].

Within the multifactorial aetiology of TMD pain, AB and SB, psychosocial factors play a significant role [6, 7]. A psychosocial factor of interest in TMD pain, AB and SB is post‐traumatic stress disorder (PTSD) [8, 9, 10, 11, 12, 13]. According to the Diagnostic and Statistical Manual of Mental Disorders (DSM‐5), PTSD is a mental health condition that can develop in a person who is directly or indirectly exposed to a traumatic event (e.g., actual or threatened death, serious injury, or sexual violence) [14]. PTSD is characterised by symptoms, such as re‐experiencing the traumatic event, avoidance of trauma‐related stimuli, negative thoughts or feelings, and trauma‐related arousal and reactivity that last for more than 1 month and cause distress or functional impairment [14].

Research on TMD pain and bruxism in patients with PTSD is limited. Evidence suggests that there could be an association between TMD pain and bruxism [15, 16]. Previous studies have investigated the prevalence of TMD pain, AB and SB in a population with PTSD and their possible associations with the type of traumatic event experience [11, 13]. These studies found that TMD pain, AB and SB were more prevalent among patients with PTSD than in the general population [11, 13]. Despite these new insights, there is still a lack of understanding of how these conditions are related to each other and to demographic and psychological variables. Insight into how these conditions are related would improve our understanding of TMD pain, AB and SB in patients with PTSD. It was hypothesized that TMD pain, AB and SB would be interconnected, and that these conditions would be associated with both PTSD and psychological variables, as well as demographic variables.

Therefore, this study aimed to investigate the direct and indirect associations between TMD pain, AB and SB, and demographic and psychological variables in patients with PTSD using network analysis. A network analysis can help understand the network that defines the interactions between the variables involved in PTSD and TMD pain, AB and SB, and could lead to new insights into possible associations between these variables, from which hypotheses could be formulated for further research and clinical applications.

## 2 Methods

### 2.1 Sample Population

The study subjects were consecutive patients referred to Psychotrauma Expertise Centre (PSYTREC) in Bilthoven, the Netherlands, by their healthcare professional for trauma‐related problems between July 2019 and June 2020. PSYTREC is a specialised center for the treatment of PTSD that uses a multi‐day program involving the combination of prolonged exposure (PE), eye movement desensitisation and reprocessing (EMDR), psychoeducation and physical activities [17]. Criteria for treatment at PSYTREC were a PTSD diagnosis according to *DSM‐5* [14] as established with the Clinician‐Administered PTSD Scale (CAPS‐5) [18, 19], a minimum age of 18 years, adequate command of the Dutch language, and no history of suicide attempts 3 months prior to treatment. All patients were instructed to complete all the assessments as part of the routine procedure. Patients included for treatment at PSYTREC who gave informed consent for the usage of their data for research were included in this study.

### 2.2 Procedure

After referral, intake sessions were scheduled, during which the diagnosis of PTSD was established by a trained clinical psychologist using the CAPS‐5. During the intake procedure, the patients also completed several questionnaires to assess various variables, including self‐reported TMD pain, AB and SB. Patients meeting the criteria for treatment at PSYTREC were asked for their informed consent for the use of their data for research purposes. The questionnaires used in this study were part of a larger set of questionnaires used for research at PSYTREC. This study was approved by the Ethics Review Board of the Academic Centre for Dentistry Amsterdam (protocol ID: 2023‐81471) and is in accordance with the Helsinki Declaration.

### 2.3 Variables

Information on TMD pain, AB, SB, insomnia, age and sex was obtained through questionnaires, whereas PTSD symptoms, mood disorders, anxiety disorders, alcohol abuse and drug addiction were assessed using diagnostic interviews.

TMD pain was defined as the self‐reported presence of function‐dependent pain in the jaw or temple area during the last 30 days, as assessed using the Dutch translation of the TMD pain screener [20, 21]. The TMD pain screener has excellent sensitivity, specificity, content validity and reliability [20]. The questionnaire consists of six items. The first item regarding the presence of pain in the jaw or temple area was scored 0–2, and the subsequent items regarding the presence of pain or stiffness in the morning and activities modifying pain in the jaw or temple area were scored 0–1. The maximum total score is 7, and TMD pain is considered present if patients have a total score of 3 or higher [20, 21].

AB and SB were assessed using an abbreviated version of the Dutch translation of the Oral Behaviours Checklist (OBC) [22]. The Dutch version of the OBC possesses excellent reliability and good validity [22]. The abbreviated OBC consists of five items about AB, and one item about SB. Items on AB are scored on a 5‐point scale (from 1 = ‘none of the time’ to 5 = ‘all of the time’), and items on SB were also scored on a 5‐point scale (from 1 = ‘none of the time’ to 5 = ‘4 to 7 nights a week’). Since there are no cut‐off scores established for the OBC, a conservative cut‐off was chosen to prevent over‐estimation; bruxism was only considered present if frequent bruxism was reported. Therefore, subject‐based AB was considered present if a patient scored 4 or higher on any of the 5 AB items, and subject‐based SB was considered present if a patient scored 4 or higher on the SB item.

Insomnia was assessed using the Dutch version of the Insomnia Severity Index (ISI) [23]. The ISI has excellent reliability and high validity [24]. The ISI consists of 7 items that are scored on a 5‐point scale (from 0 = ‘none’ to 4 = ‘very severe’). The ISI total score ranges from 0 to 28 and was used to indicate the severity of insomnia.

PTSD symptom severity was assessed using the Dutch version of the CAPS‐5 [18, 19]. The CAPS‐5 is considered the gold standard for assessing PTSD [25], and the Dutch version of the CAPS‐5 possesses high consistency and reliability for the total severity score [18]. The CAPS‐5 is a clinical interview by a trained professional and consists of 20 items. The 20 items correspond to the 20 PTSD symptoms based on DSM‐5 and are rated on a 5‐point scale for intensity (from 0 = ‘absent’ to 4 = ‘extreme’) and frequency (from 0 = ‘never’ to 4 = ‘almost daily’). The CAPS‐5 total score ranges from 0 to 80 and is used to indicate the severity of PTSD symptoms.

The presence of mood disorders, anxiety disorders, alcohol abuse and drug addiction was assessed using the Dutch version of the Mini‐International Neuropsychiatric Interview (M.I.N.I) [26]. M.I.N.I. is a reliable, valid, and structured diagnostic interview for DSM‐5 diagnoses and includes interview questions and criteria for the assessment of the presence of mood disorders, anxiety disorders, alcohol abuse and drug addiction. M.I.N.I. was developed as a short but accurate structured diagnostic interview for psychiatric diagnoses, suitable for clinical trials and epidemiology studies, and can be administered in approximately 15 min [26].

### 2.4 Statistical Analysis

To investigate and visualise both direct and indirect associations between the variables, whereas controlling for all other variables, a network analysis was performed in R statistical software (version 4.3.1, R Core Team, Vienna, Austria) [27]. A Mixed Graphical Model (MGM) [28] was estimated to evaluate the relationship between the variables using the R‐package ‘bootnet’ (version 1.5.5) [27]. The following variables were included as categorical variables: self‐reported TMD pain (‘TMD pain present’, ‘no TMD pain present’), self‐reported AB (‘AB present’, ‘no AB present’), self‐reported SB (‘SB present’, ‘no SB present’), sex (‘female’, ‘male’), mood disorders (‘mood disorder present’, ‘no mood disorder present’) and anxiety disorders (‘anxiety disorder present’, ‘no anxiety disorder present’). The following continuous variables were included: age, PTSD symptom severity and insomnia severity. Alcohol abuse (*n* = 14) and drug addiction (*n* = 7) had very low prevalence in the study population; hence, these variables could not be included in the network analysis.

Because of the relatively small sample size for network analysis and to reduce the inclusion of false positive or spurious edges, the ‘least absolute shrinkage and selection operator’ (LASSO) was applied as a regularisation technique [29, 30]. LASSO regularisation was controlled by tuning parameter *λ* (lambda). However, this parameter cannot be directly set. Therefore, this tuning parameter was selected by optimization of the Extended Bayesian Information Criterion (EBIC) by determining hyperparameter *γ* (gamma). Hyperparameter *γ* is typically set between 0 and 0.5, where a higher value is more stringent and will apply more regularisation resulting in a sparser network, and a lower value is more lenient and will apply less regularisation resulting in more edges being estimated [29]. For this network analysis, a *γ* of 0.5 was selected for higher specificity. Currently, no power analysis techniques are available for network analysis.

The associations between the variables in the estimated network were visualised using the R‐package ‘qgraph’ (version 1.9.5) [31]. The variables were visualised in the network model as nodes, linked by edges between nodes that represented the conditional dependence associations. Nodes for categorical variables are displayed as squares and nodes for continuous variables are displayed as circles. The colour of the edges indicates a positive or negative association (blue = positive association, red = negative association), and thicker and darker coloured edges between nodes indicate stronger associations.

To investigate the accuracy of the estimated network, stability checks were conducted to evaluate the robustness of the network using the bootstrapping method [27]. By repeatedly estimating a model from simulated data, bootstrapping produces a plot of 95% bootstrapped confidence intervals (CIs). Non‐parametric bootstrapping with 1000 samples was applied to estimate the accuracy of the edges. When the bootstrapped CIs of the edges are wide, they should be interpreted with caution.

## 3 Results

717 subjects gave consent for the usage of their data for research, and this group consisted of 168 male and 549 female subjects with a mean age of 39.5 ± 12.3 years. After excluding subjects with missing data, the group included in the network analysis consisted of 597 subjects, including 137 male and 460 female subjects with a mean age of 39.3 ± 12.3 years, with a range between 18 and 72 years. An independent samples *t*‐test showed no significant difference in age (*p* = 0.986) and a Chi‐square test showed no significant difference in sex (*p* = 0.496) between the excluded group due to missing data and the group included in the network analysis. For the group included in the network analysis, self‐reported TMD pain was present in 189 subjects (31.7%), AB in 289 subjects (48.4%) and SB in 245 subjects (41.0%).

**FIGURE 1:**
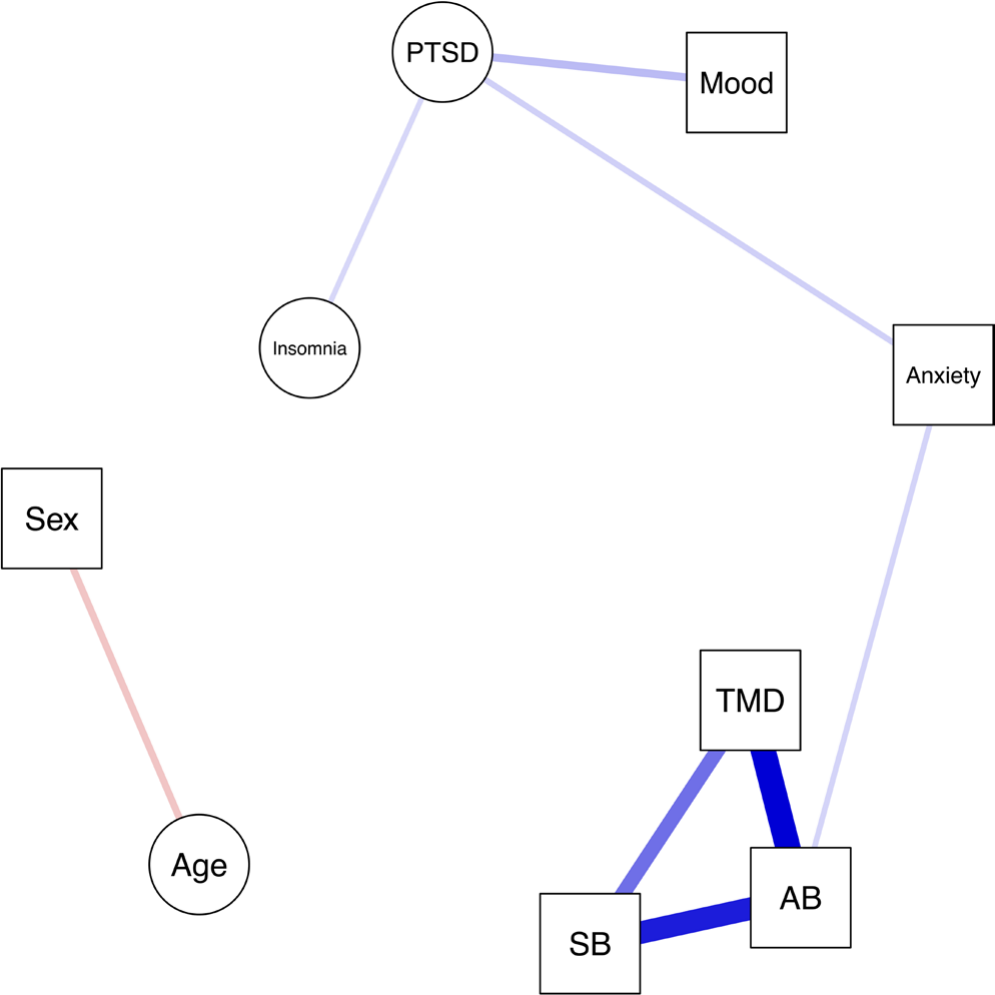
Network model of TMD pain, awake bruxism, and sleep bruxism, and demographic and psychological variables. Circles represent continuous variables; squares, categorical variables. Blue lines represent positive associations; red lines, negative associations. Thicker and darker coloured lines refer to stronger associations. TMD = self‐reported TMD pain, AB = self‐reported awake bruxism, SB = self‐reported sleep bruxism, Sex = female sex, PTSD = CAPS5 score, Mood = mood disorders, Anxiety = anxiety disorders, Insomnia = Insomnia Severity Index.

The network model is visualised in Figure 1. This network model indicates a triangle of positive edges between self‐reported TMD pain, AB and SB, indicating that patients with self‐reported TMD pain tend to have self‐reported AB and SB. In addition to a positive edge with self‐reported SB, self‐reported AB has a positive edge with anxiety disorders. PTSD symptom severity has positive edges with mood disorders, insomnia and anxiety disorders. This indicates that subjects with higher PTSD symptom severity tend to have mood disorders, anxiety disorders, and a higher insomnia severity and vice versa. The presence of anxiety disorders serves as a bridge factor connecting AB and PTSD symptom severity. In addition, the presence of anxiety disorders is a bridge factor connecting the cluster of TMD pain, AB and SB with the cluster of PTSD symptom severity, insomnia severity and the presence of mood disorders. Age and sex were not associated with other variables in the network model, but a negative node was present between age and sex, indicating that, for the study population, female subjects tended to be younger. A plot of the 95% bootstrapped CIs of this estimated network model is presented in Appendix 1.

## 4 Discussion

This study aimed to investigate the relationship between self‐reported TMD pain, AB, SB, demographic and psychological variables in a PTSD‐patient population, using network analysis. The network model showed that self‐reported TMD pain was associated with self‐reported AB and SB. Anxiety disorders served as a bridge factor, linking TMD pain, AB and SB to PTSD symptom severity, insomnia severity and mood disorders. Age and sex were associated with each other but were not linked to any other variables in this network.

The association between bruxism and TMD pain has been a topic of research in the past, resulting in mixed findings regarding both the presence and strength of associations between TMD pain and AB or SB [15, 16]. A study in an adult TMD‐patient population found an association between self‐reported AB and TMD pain in a network model [32]. These findings correspond with the current network model, in which a strong association was also observed between TMD pain and self‐reported AB. This suggests that AB may be associated with TMD pain not only in a TMD‐patient population but also in a specific population of people with PTSD. In the current network, an association was also present between self‐reported SB and TMD pain, which is in contrast to the findings of Chattrattrai et al. [32], where this association was not found within a TMD‐patient population. Hence, the association between self‐reported SB and TMD pain in patients with PTSD should be investigated further.

Prior research suggests a relationship between PTSD and TMD pain [10]. A recent case–control study also found AB to be associated with PTSD, but no association was found with SB [13]. Furthermore, a previous study on the same PTSD population found that the severity of TMD pain and both AB and SB were positively associated with the severity of PTSD symptoms [11]. In our network model, an indirect relationship between PTSD and TMD pain was observed; the severity of the PTSD symptoms was not directly associated with TMD pain, AB and SB, but rather through the presence of anxiety disorders. The literature suggests that anxiety symptoms are associated with AB, but is unclear regarding the association between anxiety symptoms and SB [33, 34]. The presence of a direct association between AB and anxiety disorders and the absence of a direct association between SB and anxiety disorders could support the notion that AB may be more strongly related to psychosocial factors, such as anxiety, than SB [7].

Anxiety disorders are frequently comorbid with PTSD [35, 36]. Previously, PTSD has been classified as an anxiety‐related disorder in the previous edition of the Diagnostic and Statistical Manual of Mental Disorders (DSM‐IV), whereas it now falls under the new header of trauma/stressor‐related disorders in the DSM‐5 [14, 37]. In our network, anxiety disorders serve as a bridging factor between the cluster of TMD pain and bruxism on the one hand and the cluster of psychological factors, including PTSD severity, insomnia and mood disorders on the other. PTSD and anxiety disorders share common pathophysiological features, such as changes in adrenal, autonomic, structural and functional characteristics [38]. One of these pathological features common to both PTSD and anxiety disorders is changes in the hypothalamic–pituitary–adrenal (HPA) axis, which is integral in the regulation of the stress response through cortisol [38]. TMD is associated with anxiety [39], and within subjects with TMD pain, earlier research also pointed out that anxiety may contribute to the chronic upregulation of the HPA axis [40]. Furthermore, higher levels of cortisol are associated with bruxism [41]. This could imply that changes in the HPA axis could be a possible common underlying mechanism of the associations in the current network model between self‐reported TMD pain, AB and SB on the one hand and psychosocial factors on the other hand, which are connected by the presence of anxiety disorders. Future studies should investigate the role of anxiety disorders and possible common underlying mechanisms in patients with comorbid PTSD, TMD pain and bruxism.

PTSD is more prevalent in females than in males [35, 42]. Furthermore, previous studies showed that females have a higher PTSD prevalence with younger age, while males showed a higher PTSD prevalence with older age [35, 42]. This study had a high female‐to‐male ratio, and within this network model, age and sex were negatively associated, suggesting that female subjects tended to be younger, which is in agreement with the literature. Age and sex were not associated with other variables included in the network analysis. In the general population, the peak occurrence of TMD pain is between 20 and 40 years of age, and TMD pain is more prevalent in women than in men [43, 44]. The mean age of the group included in the analysis was 39.3 ± 12.3 years old, which overlaps with the peak occurrence rate of TMD in the general population. This could provide a possible explanation for the absence of an association between age and TMD pain in this network model. The reason for the female preponderance in TMD prevalence is not completely clear, but factors such as hormonal influences have been hypothesized [45]. Contrary to the general population, no association was found in this network between sex and TMD pain, which could suggest that within subjects with PTSD, other influences unrelated to sex could have a more prominent role in the multifactorial aetiology of TMD pain. Further research is needed to investigate and explain these findings.

Research into the associations between PTSD and TMD pain and between PTSD and bruxism is relatively sparse and is mainly performed in TMD pain or bruxism populations. This highlights a strength of this study, as it was conducted with a PTSD‐patient population, providing insight from a PTSD perspective. Furthermore, the available literature on TMD pain or bruxism within PTSD populations mainly involves subjects exposed to war [12], whereas this study included individuals exposed to different types of traumatic events (e.g., physical violence, serious accidents, sexual abuse) [11]. Moreover, to the best of our knowledge, this is the first study to investigate TMD pain, AB and SB in PTSD using a network analysis to determine associations between these and several other variables of interest. The benefit of using such an analysis method over univariate or multivariate regression models is that all variables are considered as both predictor and outcome variables. This enables visualisation of the structure of the data and identification of direct and indirect associations, while controlling for other variables.

A limitation of this research is its cross‐sectional study design; hence, causal relationships cannot be drawn. A further limitation of this study was the use of self‐reporting in the assessment of TMD pain, AB and SB. The gold standard would be to use a clinical examination according to the diagnostic criteria for temporomandibular disorders (DC/TMD) [2]. Despite the excellent reliability and good validity of the OBC [22], self‐reporting of AB and SB could only establish a diagnosis of possible bruxism [4]. To provide a more definite assessment of bruxism, ecological momentary assessment (for AB) or electromyography/polysomnography (for SB) could be used for a higher‐level assessment [4]. Due to logistic constraints regarding the multi‐day treatment at PSYTREC, these alternative assessment methods for TMD pain, AB and SB could not be employed.

An additional limitation of this study is the absence of data on potential aggravating or alleviating factors for TMD pain and bruxism, such as certain comorbidities for these conditions (e.g., rheumatoid arthritis) or the use of specific psychopharmaceuticals that could influence chronic pain, bruxism, or sleep (e.g., tricyclic antidepressants, selective serotonin reuptake inhibitors, benzodiazepines). Although some psychological comorbidities were included, data on other health conditions and medication use were not available due to the study's embedding in routine clinical care and the constraints on data collection at PSYTREC. Moreover, the relatively small sample size restricted the number of variables that could be incorporated into the network analysis. Future research could specifically examine the role of comorbidities and psychopharmaceuticals in the context of TMD pain and bruxism in patients with PTSD.

This network analysis revealed the significant role of anxiety disorders as a bridging factor between physical symptoms, such as TMD pain, AB and SB, and psychological factors, such as PTSD severity, mood disorders and insomnia in patients with PTSD. Clinically, the findings of this study underscore the necessity of an integrated management approach and the importance of psychological screening for anxiety and related disorders in patients with TMD pain and bruxism. The identified positive associations between PTSD symptom severity, mood disorders, and insomnia, along with their connection with physical symptoms such as bruxism and TMD pain through anxiety disorders, suggest the need for targeted interventions. For patients exhibiting this cluster of symptoms, interventions that concurrently address PTSD, anxiety disorders, mood disorders and insomnia are recommended. Therapeutic strategies such as cognitive‐behavioural therapy (CBT) or EMDR therapy, which can address multiple psychological conditions simultaneously, might be particularly beneficial in this patient population [46]. A recent study has demonstrated a decrease in self‐reported TMD pain, AB and SB in a PTSD population after PTSD treatment [47]. More research is recommended to further investigate the effect of targeted interventions for PTSD and comorbid conditions on TMD pain and bruxism in individuals with PTSD.

This study aimed to assess the possible associations of TMD pain and bruxism with PTSD. Several associations were identified in the network model. The findings of this study highlight the intricate interplay between TMD pain, AB and SB and psychological conditions, with anxiety as a bridging factor. Among subjects with PTSD, TMD pain, AB and SB were strongly associated with each other. These new insights could provide a basis for future research to broaden the understanding of these conditions, with the ultimate goal of providing better personalised care for patients with TMD pain, AB, SB and comorbid PTSD.

## Author Contributions

Joey Chung contributed to the conception, design, and data interpretation; performed all statistical analyses; and drafted and critically revised the manuscript. Wendy Knibbe contributed to conception, design and data interpretation, and critically revised the manuscript. Thiprawee Chattrattrai contributed to design and data interpretation, performed all statistical analysis, and critically revised the manuscript. Ad de Jongh contributed to conception, data acquisition and data interpretation, and critically revised the manuscript. Frank Lobbezoo contributed to conception, design and data interpretation, and critically revised the manuscript. All authors gave their final approval and agreed to be accountable for all aspects of this work.

## Conflicts of Interest

Joey Chung, Wendy Knibbe, and Thiprawee Chattrattrai have nothing to disclose. Ad de Jongh receives income from published books on EMDR therapy and for the training of post‐doctoral professionals in this method. Frank Lobbezoo receives research grants from SomnoMed, Sunstar Suisse, S.A., Vivisol‐Resmed, Airway Management, and Health Holland, which are unrelated to this paper. Frank Lobbezoo is an unsalaried member of the Academic Advisory Board of Sunstar Suisse S.A. for GrindCare.

## Peer Review

The peer review history for this article is available at https://www.webofscience.com/api/gateway/wos/peer‐review/10.1111/joor.14007.

## Data Availability

The data that support the findings of this study are available on request from the corresponding author [Joey Chung; j.c.l.chung@acta.nl]. The data are not publicly available due to information that could compromise the privacy of research participants.

## Appendix 1

Bootstrapped confidence intervals (CIs) of the estimated network model. The strongest edge of the plot is showed on the first line of the plot. Overlapping CIs indicate that those edges are not significantly different. Edges displaying broad CIs should be cautiously interpreted.
